# Inflammation and regeneration in cross-organs

**DOI:** 10.1186/s41232-016-0023-4

**Published:** 2016-10-05

**Authors:** Seiji Okada, Toru Ogata

**Affiliations:** 1 Department of Advanced Initiatives, Graduate School of Medical Sciences, Kyushu University, 3-1-1 Maidashi, Higashi-ku, Fukuoka, 812-8582 Japan; 2 Department of Rehabilitation for the Movement Functions, Research Institute, National Rehabilitation Center for Persons with Disabilities, Saitama, Japan

**Keywords:** Inflammation, Tissue repair, Regeneration

The inflammation that accompanies injury and disease is a defense reaction, constituting an important part of the natural innate immune response. In any organ, inflammation is necessary for the healing process to reduce the size of a damaged or necrotic area and replace it with new tissue. When the necrotic area is replaced by new functional tissue, it is called regeneration; when it is instead replaced with abnormal scar tissue with prolonged inflammation, it is called fibrosis. Most organs will heal using a mixture of both mechanisms, and the inflammatory response is closely associated with these processes. The inflammation itself can support tissue protection and regeneration; however, it can also lead to further damage and dysfunction, like a double-edged sword.

It is often difficult to recognize whether inflammation is harmful or beneficial for organ regeneration, since inflammatory reactions can be clinically, pathologically, and physiologically variable, depending on the cause and stage. In addition, a variety of inflammatory mediators finely orchestrate inflammation through extremely complicated cellular interactions, which makes inflammation nearly impossible to understand completely. Inflammation can be either therapeutic or exacerbating in different situations or organs, even when executed in the exact same manner.

However, the act of mixing different insights from mutually irrelevant fields (organs or disease) can occasionally lead to the development of new insights. Indeed, in this manner, I have identified a new mechanism in the healing process following traumatic central nervous system (CNS) injury by examining the skin wound healing process [1]. Keratinocyte migration via Stat3 signaling was found to be responsible for skin wound healing, and Stat3 deficiency results in compromised healing [2]. Similar to this, Stat3 was found to be essential after spinal cord injury for the migration of astrocytes, the most abundant cell population in the CNS.

We invited the leading researchers on inflammatory disease in several different organs to participate in this review. Dr. Shichita’s group summarized their recent findings regarding the role of post-ischemic inflammation in ischemic stroke pathology. I reviewed a pathophysiological role of acute inflammation in spinal cord injury. Dr. Yamada’s group discussed the relationship between inflammatory cells and lung injury/repair mechanisms. Dr. Miyajima expounded on new techniques for examining the process of liver regeneration and its mechanisms. Dr. Tamai reviewed the role of high-mobility group box 1 (HMGB1) and the associated functional cells in skin regeneration. I would like to express my gratitude to the distinguished researchers who contributed to this special issue. I sincerely hope that these review articles will offer some novel insight beyond the boundaries of the organ to scientists in the field of inflammation and regeneration.
